# Genetic engineering of a thermophilic acetogen, *Moorella thermoacetica* Y72, to enable acetoin production

**DOI:** 10.3389/fbioe.2024.1398467

**Published:** 2024-05-15

**Authors:** Junya Kato, Tatsuya Fujii, Setsu Kato, Keisuke Wada, Masahiro Watanabe, Yusuke Nakamichi, Yoshiteru Aoi, Tomotake Morita, Katsuji Murakami, Yutaka Nakashimada

**Affiliations:** ^1^ Graduate School of Integrated Sciences for Life, Hiroshima University, Higashihiroshima, Hiroshima, Japan; ^2^ National Institute of Advanced Industrial Science and Technology (AIST), Higashihiroshima, Hiroshima, Japan; ^3^ National Institute of Advanced Industrial Science and Technology (AIST), Ibaraki, Japan

**Keywords:** thermophilic acetogen, genetic engineering, C4 chemicals, acetoin, biomass, gaseous substrates

## Abstract

Acetogens are among the key microorganisms involved in the bioproduction of commodity chemicals from diverse carbon resources, such as biomass and waste gas. Thermophilic acetogens are particularly attractive because fermentation at higher temperatures offers multiple advantages. However, the main target product is acetic acid. Therefore, it is necessary to reshape metabolism using genetic engineering to produce the desired chemicals with varied carbon lengths. Although such metabolic engineering has been hampered by the difficulty involved in genetic modification, a model thermophilic acetogen, *M. thermoacetica* ATCC 39073, is the case with a few successful cases of C2 and C3 compound production, other than acetate. This brief report attempts to expand the product spectrum to include C4 compounds by using strain Y72 of *Moorella thermoacetica*. Strain Y72 is a strain related to the type strain ATCC 39073 and has been reported to have a less stringent restriction-modification system, which could alleviate the cumbersome transformation process. A simplified procedure successfully introduced a key enzyme for acetoin (a C4 chemical) production, and the resulting strains produced acetoin from sugars and gaseous substrates. The culture profile revealed varied acetoin yields depending on the type of substrate and culture conditions, implying the need for further engineering in the future. Thus, the use of a user-friendly chassis could benefit the genetic engineering of *M. thermoacetica*.

## 1 Introduction

One of the global challenges in this modern era is establishing a sustainable society free from fossil resource-dependent industries. A key factor is the utilization of sustainable resources, such as biomass, and recycling of used materials. In this context, one of the promising approaches will be the biological conversion of these resources to conventional chemicals.

Acetogens are a group of microorganisms with unique and versatile metabolic pathways that catabolize both biomass-derived sugars and gaseous substrates to produce mainly acetic acid. Near stoichiometric conversion of hexose sugar to acetic acid (one hexose molecule is converted to three acetic acid molecules) is possible, inferring great metabolic potential (Drake and Daniel, 2004). Moreover, the gaseous substrates include hydrogen (H_2_), carbon monoxide (CO), and carbon dioxides (CO_2_), which can be derived not only from waste gases but also from the gasification of organic materials, including recalcitrant resources, such as lignin from biomass. Therefore, acetogens are promising candidates for biological catalysts that produce conventional chemicals by utilizing sustainable resources. In particular, thermophilic acetogens have multiple advantages over others, such as low risk of contamination, lower cooling cost, faster reaction speed, and simplified product recovery (Taylor et al., 2009; Abdel-Banat et al., 2010; Redl et al., 2017; Kato et al., 2021).

For the practical application of thermophilic acetogens, it is essential to produce chemicals that are more diverse than acetate. One method is to screen for new strains with the metabolic capacity to produce these chemicals. Another method is genetic engineering of thermophilic acetogens to introduce new metabolic pathways and tune innate pathways. However, acetogens are known for their inaccessibility in genetic modification (Bourgade et al., 2021). To date, the most successful cases of metabolic engineering to produce other chemicals have been achieved using the model thermophilic acetogen *Moorella thermoacetica* ATCC 39073. These cases are limited to the production of C2 and C3 compounds, such as ethanol (C2), lactate, acetone, and isopropanol (C3) (Iwasaki et al., 2017; Rahayu et al., 2017; Kato et al., 2021; Kato et al., 2024). Metabolic engineering has been accomplished by overcoming genetic barriers via DNA premethylation (Kita et al., 2013a). However, the method is cumbersome, enabling only a small set of engineered strains obtained.

Previously, another strain of *M. thermoacetica*, Y72, was reported to be genetically more accessible than ATCC 39073, because of its less strict restriction-modification system (Kita et al., 2013b). The genome is closely related to ATCC 39073, according to the draft genome sequence, with a similar size and the same GC content (56%) (Tsukahara et al., 2014). Phylogenetic analysis revealed that Y72 belongs to the same clade as ATCC 39073, and an average nucleotide analysis further supported the similarity of the two genomes (Redl et al., 2019). Despite this similarity, strain Y72 possesses fewer genes for the restriction-modification system than ATCC 39073, which is assumed to be the reason why Y72 shows high transformation efficiency (Tsukahara et al., 2014). Therefore, Y72 may be useful as an alternative strain for the model ATCC 39073 for metabolic engineering in introducing new pathways.

Acetoin (3-hydroxy-2-butanone) is an important C4 chemical in the food, medical, and chemical industries and was selected by the U.S. Department of Energy (DOE) as one of the 30 compounds that require prioritized production from biomass sugars and syngas (Werpy et al., 2004). However, genetic engineering of thermophilic acetogens for acetoin production has not yet been reported. Moreover, no studies have reported the production of C4 chemicals using thermophilic acetogens.

In this study, metabolic engineering for the C4 chemical production by a thermophilic acetogen was attempted by taking advantage of the strain *M. thermoacetica* Y72, with acetoin as the target product. Furthermore, engineered strains were evaluated as the place to study for chassis development.

## 2 Methods

### 2.1 Strains and culture conditions

A *pyrF* knockout strain of *M. thermoacetica* Y72 was used in this study for genetic transformation (Kita et al., 2013b). The *pyrF* knockout strain and the derivative strains were cultured in serum bottles under anaerobic conditions at 55°C unless otherwise noted.

The culture medium was prepared by mixing 1.0 g of NH_4_Cl, 0.1 g of KCl, 0.2 g of MgSO_4_·7H_2_O, 0.8 g of NaCl, 0.1 g of KH_2_PO_4_, 0.02 g of CaCl_2_·2H_2_O, 2.0 g of NaHCO_3_, 10 mL of trace elements, 10 mL of Wolfe’s vitamin solution, and 1.0 mg of resazurin per liter of water (Tanner, 1989; Tanner et al., 1993). After adjusting the pH to 6.9 with 6N HCl, the medium was prepared anaerobically by boiling and cooling under an N_2_/CO_2_ (80/20 ratio) atmosphere. After cooling, the medium was dispensed into 125-mL serum bottles under N_2_/CO_2_ atmosphere. Serum bottles were crimp-sealed and autoclaved. Yeast extract and l-cysteine·H_2_O stock solutions were prepared separately and added to the medium before inoculation to reach a final concentration of 1.0 and 1.2 g/L, respectively. The total medium volume was adjusted to 50 mL. When sugars were supplied as substrates, stock solutions were prepared separately and added to the medium at a final concentration of 10–11 mM. Gaseous substrates were added to the headspace of the bottles by adjusting the partial pressure during injection. When syngas was used, CO and H_2_ were injected at 0.04 MPa, respectively. When CO_2_+H_2_ gas was used, the headspace was replaced with a mixed gas (CO_2_:H_2_ = 8:2) and adjusted to 0.2 MPa. The seed culture was grown on fructose, and used for sugar-based cultures and CO_2_+H_2_ gas-based culture (5% inoculum). In the case of syngas-based culture, the sugar-based seed culture was subsequently inoculated to a syngas-containing medium, which was used as the seed culture. All the inoculum was from the exponential phase.

### 2.2 Plasmid construction and transformation

The DNA sequence of the bsALDC from *Bacillus subtilis* IPE5-4 was codon-optimized for *M. thermoacetica* and synthesized (Genewiz). The PCR primers used for the following procedure are listed in Supplementary Table S1. Plasmid pHM23 for the introduction of *bsALDC* into *pduL2* region was constructed as follows. Primers JK109 and JK110 were used for PCR amplification of the *bsALDC* sequence. The amplified fragment was cloned using the In-Fusion HD Cloning kit (Takara) into the PCR-amplified plasmid using primer sets JK52 and JK71 with pK18-Δ*pduL2*::*ldh* as the template (Iwasaki et al., 2017). The plasmid pK18-Δ*pyrF*::*bsALDC*, which was used to introduce *bsALDC* into *pyrF* region, was constructed in the same manner. Primers, bsALDC_FW bsALDC_RV, and a template, pHM23, were used for the insert. Primers, Vec_FW and Vec_RV, and a template, pK18-kan2 (Iwasaki et al., 2013), were used for the vector. The constructed plasmids were introduced into *M. thermoacetica* Y72 following an electroporation protocol described previously, except for omitting the DNA premethylation procedure (Kita et al., 2013b; Kato et al., 2021). Homologous recombination was confirmed via PCR amplification using the primer sets pduL2_check_FW and pduL2_check_Rv for the *pduL2* locus, and pyrF72_upF and pyrF_downR for the *pyrF* locus.

### 2.3 Analytical methods

To analyze cell growth and products, 1 mL of the culture was sampled. Cell growth was determined by measuring the optical density (OD) at 600 nm. The products in the supernatant were analyzed via HPLC (LC-2000 Plus HPLC; Jasco, Tokyo, Japan) using a refractive index detector (RI-2031 Plus; Jasco). Shodex RSpak KC-811 column (Showa Denko, Kanagawa, Japan) and Shodex RSpak KC-G guard column (Showa Denko) were used. The column temperature was maintained at 60°C, and the isocratic method was used in 0.1% (v/v) phosphoric acid at a flow rate of 0.7 mL/min.

## 3 Results

### 3.1 Design of metabolic pathway for acetoin production in *M. thermoacetica*

One route for the biological production of acetoin is decarboxylation of acetolactate (Maina et al., 2022). Acetolactate is a primary metabolic intermediate in the synthesis of branched-chain amino acids, such as valine, leucine, and isoleucine. Because *M. thermoacetica* Y72 can grow in the basal medium which does not contain these amino acids derived from acetolactate, the strain Y72 should be able to synthesize acetolactate by itself (Kita et al., 2013b). Furthermore, acetolactate is synthesized from pyruvate by acetolactate synthase, which is involved in both heterotrophic and autotrophic growth of acetogens (Schuchmann and Muller, 2014). The genome contains genes encoding acetolactate synthase, whose amino acid sequence is highly similar to those in the ATCC 39073 (similarity >99%). Therefore, the introduction of a decarboxylase specific to acetolactate could enable acetoin production in the homoacetogen *M. thermoacetica* Y72.

**FIGURE 1:**
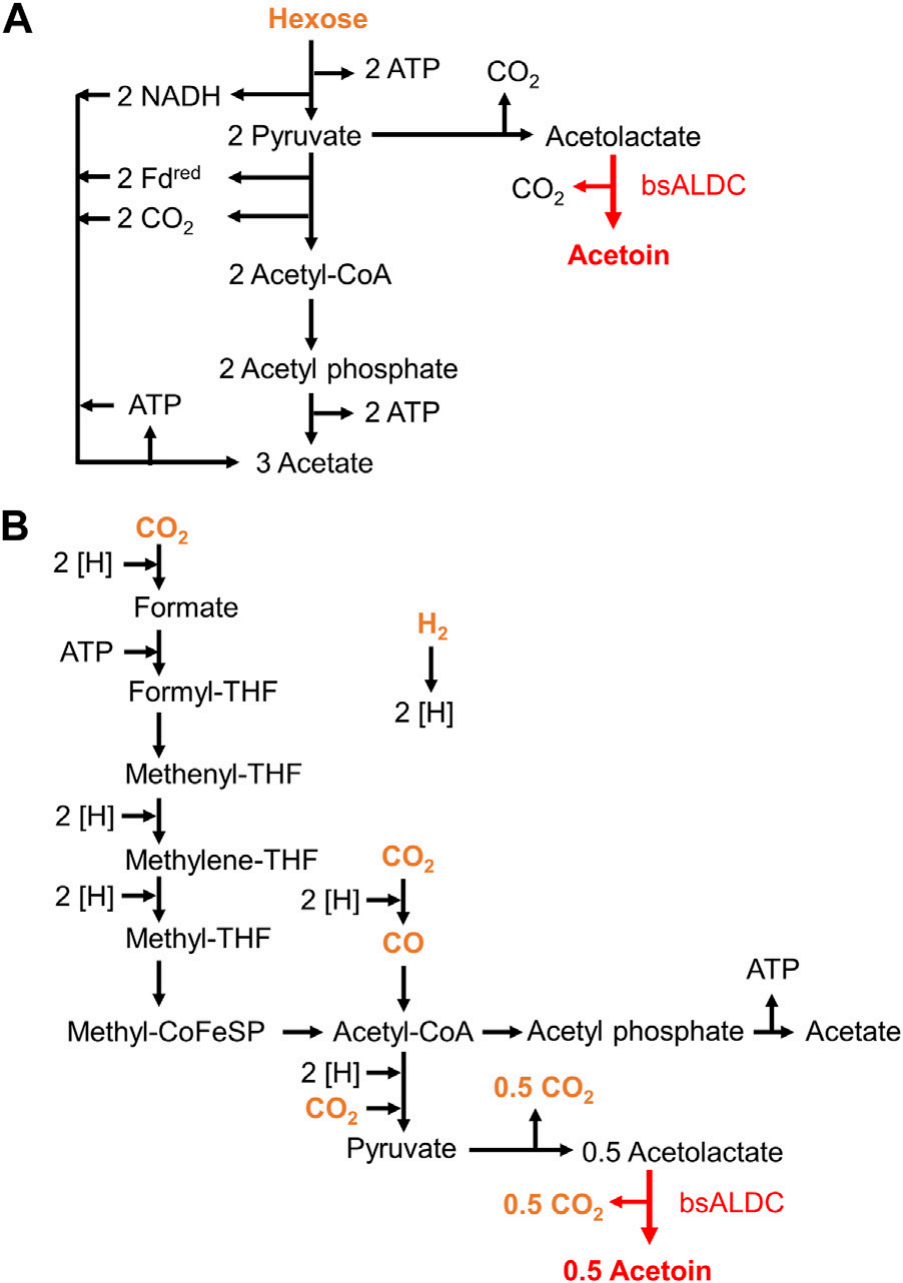
Design of metabolic pathways for acetoin production in *Moorella thermoacetica*. Pathways for acetate and acetoin production from sugars **(A)** and gaseous substrates **(B)**. The substrates are colored orange, and the key enzymatic reaction of bsALDC for acetoin production is shown in red.

As shown in Figure 1A, one hexose sugar molecule can be converted into one acetoin molecule by the introduction of acetolactate decarboxylase. In this metabolic pathway, two adenosine triphosphate (ATP) molecules are involved in glycolysis and support cellular energy production. Notably, the two NADH molecules were produced by reducing NAD^+^ and were not oxidized or recycled. Such a redox imbalance causes metabolic arrest, and it is important to maintain acetate production to some extent for NAD^+^/NADH maintenance.

In contrast, on gaseous substrates, two molecules of CO_2_ or CO were converted into one acetyl-CoA molecule. Acetyl-CoA is converted to acetate, coupled with substrate-level phosphorylation (SLP), for ATP formation in acetogenesis, which would cover a large portion of the cellular energy supply for autotrophic growth. When acetoin is produced, two acetyl-CoA molecules are used for one acetoin molecule (Figure 1B). As a result, the acetyl-CoA molecules that are supposed to be used for SLP are lost, resulting in an insufficient ATP supply. It is important to maintain a certain level of acetate production to achieve sufficient SLP. Thus, these strains were designed to preserve acetate production.

Acetolactate decarboxylase was selected for heterologous expression analysis. Because the enzyme must be tolerant to the elevated temperatures in thermophiles, bsALDC from *B. subtilis* IPE5-4 was selected (Jia et al., 2017). Enzymatic activity was maintained at high levels at the optimum growth temperature of *M. thermoacetica* (Drake and Daniel, 2004).

### 3.2 Free of customized DNA modification for the introduction of bsALDC-encoding gene into *M. thermoacetica* Y72

**FIGURE 2:**
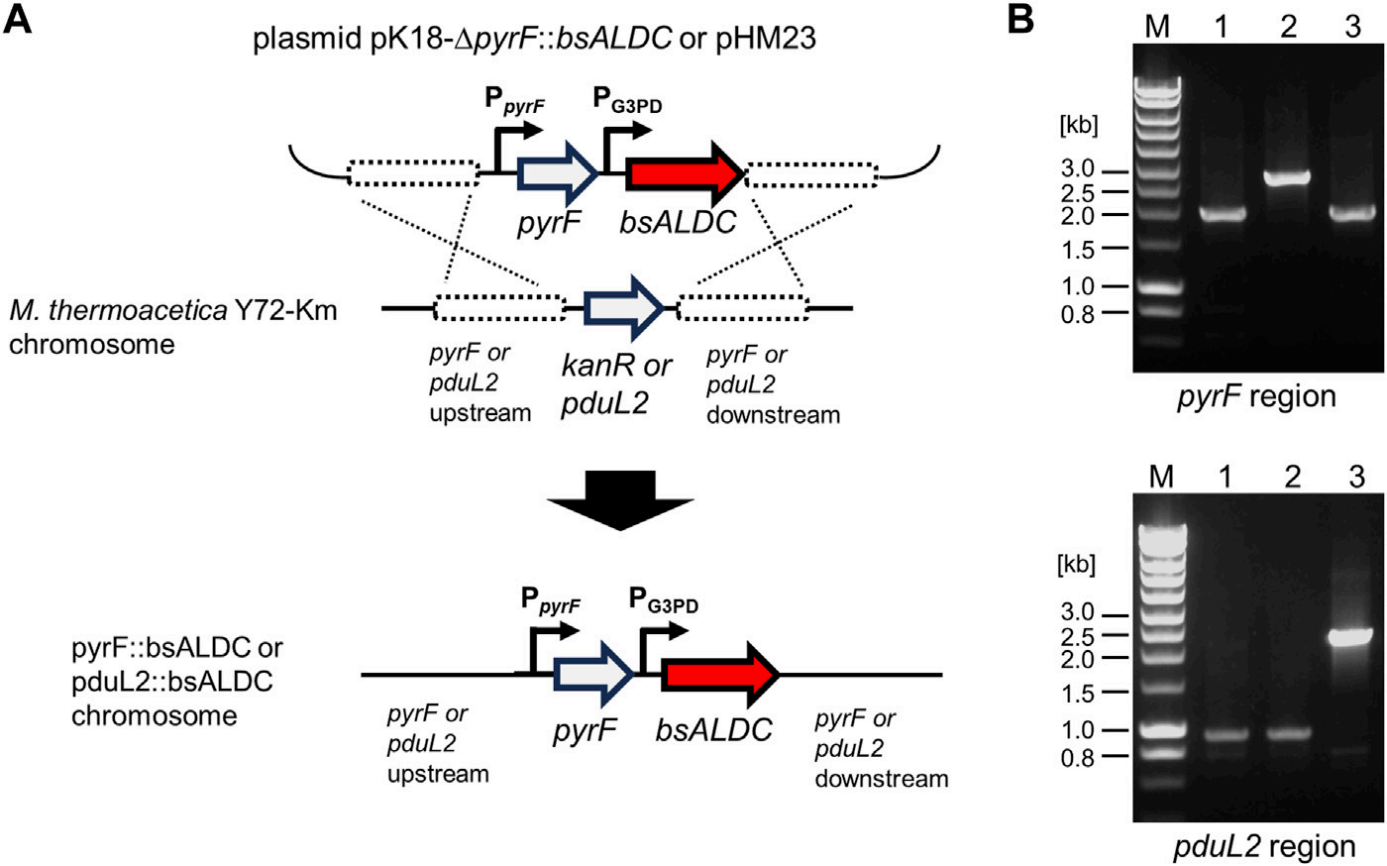
DNA construction for introducing *bsALDC* into the chromosome of *Moorella thermoacetica* Y72 by homologous recombination. **(A)** shows the schematic representation of the constructed plasmids and the homologous recombination events in the *pyrF* or *pduL2* region. **(B)** shows the agarose gel electrophoresis following PCR amplification of the *pyrF* or *pduL2* region. The size shifts of the amplified DNA confirmed the successful introduction of the *bsALDC* constructs in the *pyrF* or *pduL2* region. 1.9 kb of the *pyrF* region was shifted to 2.7, and 0.9 kb of the *pduL2* region was shifted to 2.3 kb. M, DNA size marker; lane 1, the Y72-Km strain; lane 2, the Y72-*pyrF*::*bsALDC* strain; lane 3, the Y72-*pduL2*::*bsALDC* strain.

To heterologously express *bsALDC* in *M. thermoacetica* Y72, the coding nucleotide sequence was codon-optimized and placed under the constitutive glycerol-3-phosphate dehydrogenase (G3PD) promoter. The plasmid DNA construct was designed to introduce the promoter-gene set (P_G3PD_-*bsALDC*), either at the *pyrF* or *pduL2* locus of the chromosome, by homologous recombination. The P_G3PD_-*bsALDC* was placed in homologous regions upstream and downstream of *pyrF* and *pduL2* (Figure 2A). *pyrF* gene was used as a selection marker to transform the *pyrF*-knockout strain, which is uracil auxotroph (Kita et al., 2013b). When the P_G3PD_-*bsALDC* was introduced into the *pyrF* locus, the acetate production pathway remained intact. When the P_G3PD_-*bsALDC* was introduced into the *pduL2* locus, acetate production was lowered due to the disruption of one of the two genes encoding phosphoacetyl transferase.

The resulting plasmids were introduced into *M. thermoacetica* Y72 without any customized DNA modifications. Despite skipping the modification step, the target strains were successfully obtained (Figure 2B).

### 3.3 Acetoin production from sugars

**FIGURE 3:**
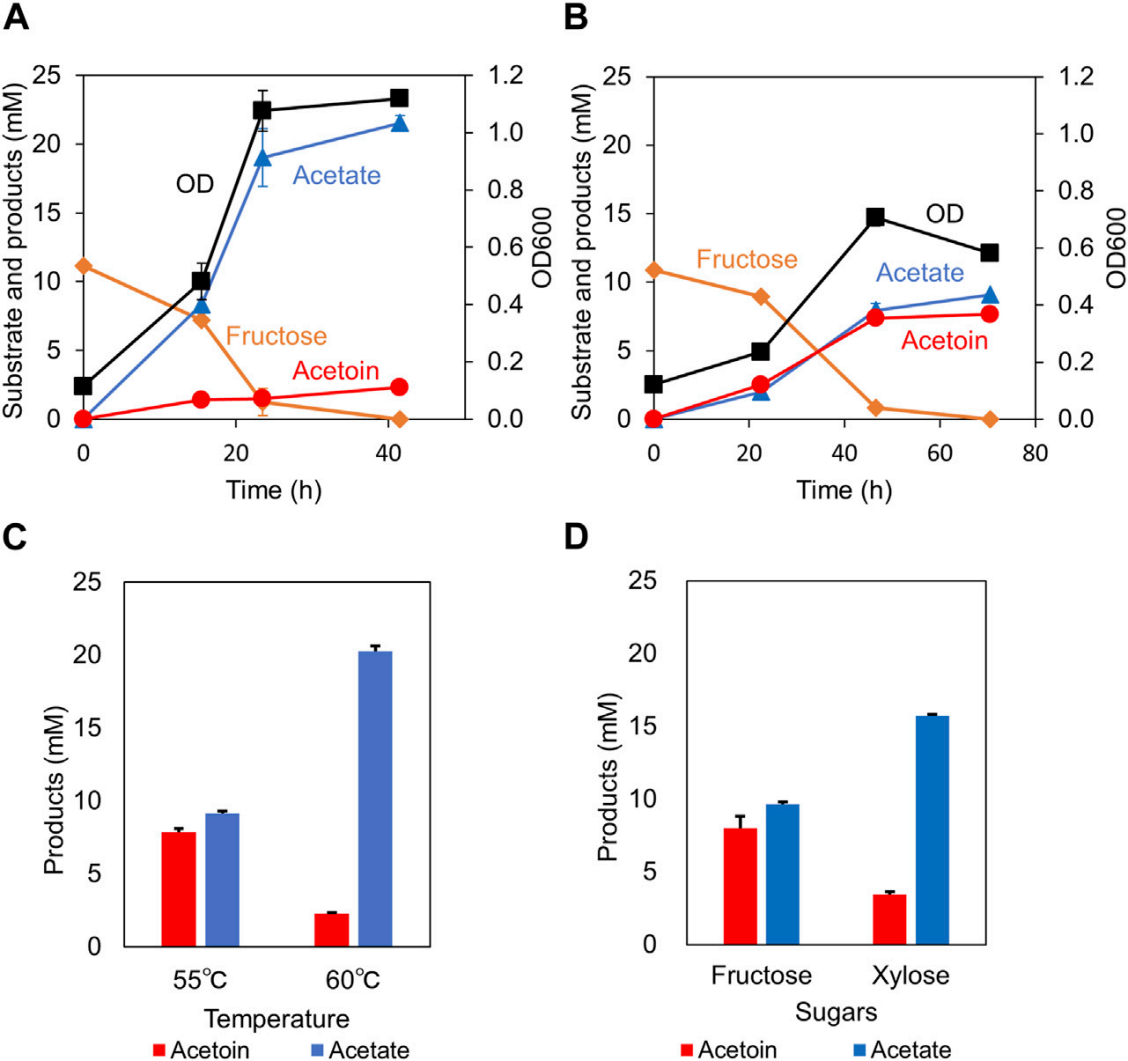
Acetoin production from sugars. The cell growth (OD), substrate consumption, acetate and acetoin productions were monitored over time in the fructose-supplemented culture. The Y72-*pyrF*::*bsALDC* **(A)** and the Y72-*pduL2*::*bsALDC* **(B)** strains are shown, respectively. **(C)** The acetoin and acetate production yields by the Y72-*pduL2::bsALDC* strain from fructose were compared between 55°C and 60°C. **(D)** The acetoin and acetate production yields by the Y72-*pduL2*::*bsALDC* strain were compared between hexose (fructose) and pentose (xylose) sugars. Data are presented as the mean with the SDs of three biological replicates. Some error bars are smaller than the symbols of data plots.

Two engineered strains containing bsALDC were tested for acetoin production from sugars. A hexose sugar, fructose, was used for the fermentation. First, a strain with an intact acetate production pathway, Y72-*pyrF*::*bsALDC*, was cultured (Figure 3A). The strain grew with two end-products in the culture supernatant. The major product was acetate, accompanied by much less acetoin. After consuming all the supplemented fructose (11.1 mM), the acetate concentration reached 21.5 mM and the acetoin concentration reached 2.3 mM. As a result, 0.21 mol-acetoin/mol-fructose was obtained.

Next, the Y72-*pduL2*::*bsALDC* strain, with a deficient acetate production pathway (*pduL2* knockout), was cultured under the same conditions (Figure 3B). The strain grew with two end-products of different profiles. The amount of acetoin was much higher, reaching 7.7 mM, whereas the acetate production decerased to 9.1 mM. The tuning of carbon flow by *pduL2* knockout was effective in enhancing the target product, acetoin. The maximum cell growth was lower than that of the Y72-*pyrF*::*bsALDC* strain, most likely because of lowered ATP production coupled with acetate production. It is known that the cellular biomass amount is correlated with the energy production (Bauchop and Elsden, 1960).

Using the Y72-*pduL2*::*bsALDC* strain, a few additional features were characterized. As the optimum temperature for the *M. thermoacetica* growth is between 55°C and 60°C, the effect of temperature was compared in this range (Figure 3C). The acetoin yield, 0.72 mol-acetoin/mol-fructose at 55°C, was lowered to 0.22 mol-acetoin/mol-fructose at 60°C. A different sugar, xylose (pentose), was tested instead of fructose (hexose) (Figure 3D). Acetoin was produced from xylose at the yield of 0.34 mol-acetoin/mol-xylose at 55°C, which is lower than that from fructose by half. Acetoin yield tended to decrease in both cases.

### 3.4 Acetoin production from gaseous substrates

Following the demonstration of acetoin production from sugars, acetoin production from gaseous substrates was tested. We selected syngas (CO, H_2_ and CO_2_) and CO_2_+H_2_ gas, because the autotrophic growth and chemical production would be different based on the energy source (Hu et al., 2016); CO provides higher energy than H_2_. While syngas contains both CO and H_2_ as energy sources, CO_2_+H_2_ gas contains only H_2_.

**FIGURE 4:**
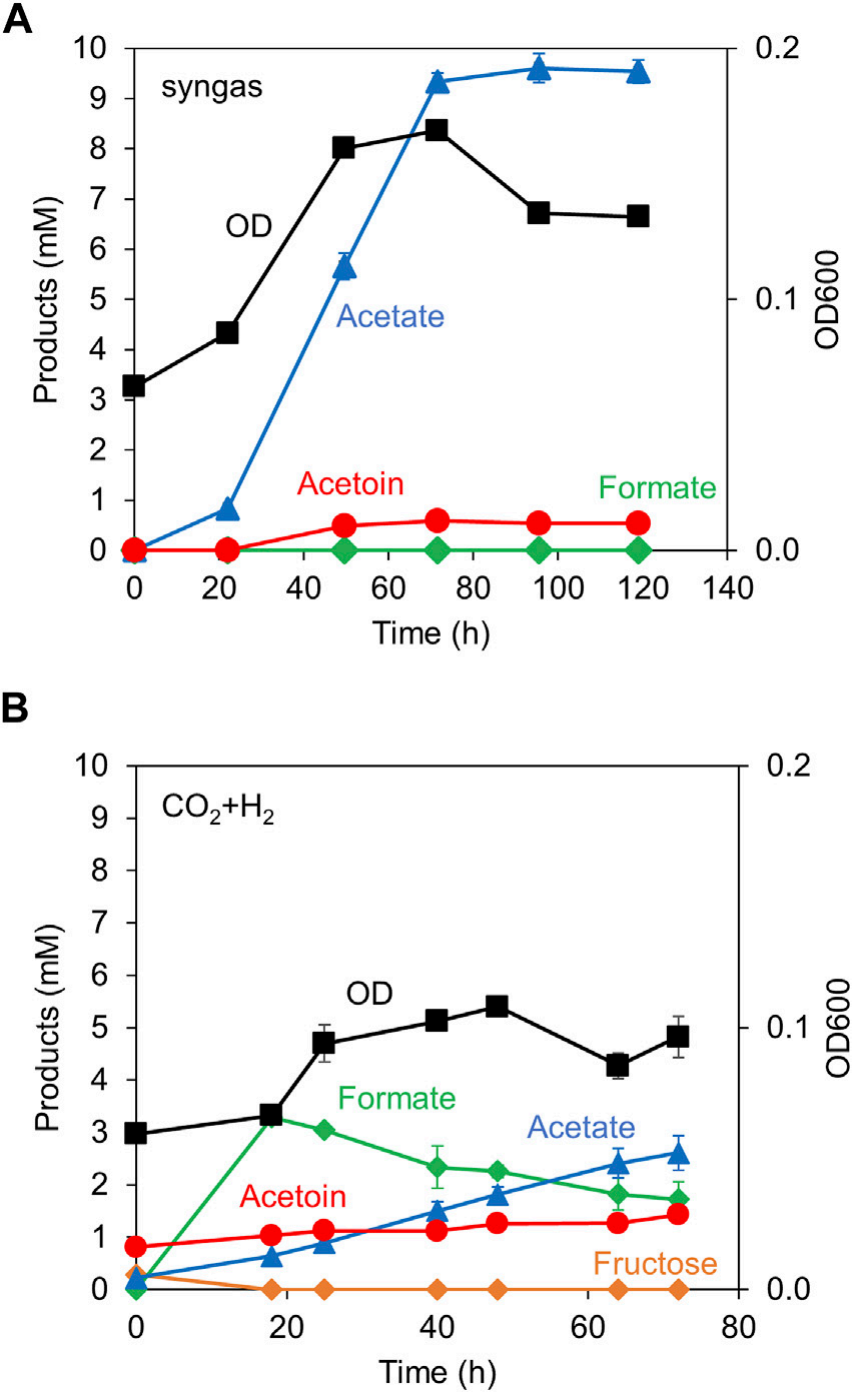
Acetoin production from gaseous substrates. The cell growth (OD) and the production of acetate, acetoin, and formate were monitored over time. Syngas **(A)** or CO_2_+H_2_ gas **(B)** was supplemented in the head space of culture vials. Data are presented as the mean with SDs of three **(A)** and two **(B)** biological replicates, respectively. Some error bars are smaller than the symbols of data plots. Fructose concentration was shown in **(B)** to indicate its absence and the residual amount from the seed culture.

When the Y72-*pduL2*::*bsALDC* strain was cultured in the syngas, the strain grew with abundant acetate production (Figure 4A). Acetoin production was 20-fold less than acetate production (0.5 mM vs 9.5 mM). Formate, an intermediate in the metabolism of CO_2_, was not observed, indicating that the sufficient ATP was provided. ATP was required to process formate to formyl-THF (Figure 1B). However, when the same strain was cultured in CO_2_+H_2_ gas, the OD value remained almost constant, indicating that the cells did not grow. As the major metabolite, a significant amount of formate was accumulated, indicating that ATP was in short supply (Figure 4B), consistent with the growth profile. Acetate was gradually accumulated (2.4 mM). Acetoin was also produced and accumulated gradually; however, the amount remained limited (0.6 mM), as in the syngas culture.

## 4 Discussion

This brief report describes the metabolic conversion of a thermophilic homoacetogen, *M. thermoacetica* Y72, using genetic engineering. The strain originally produced only acetate (Kita et al., 2013b), and the successful introduction of a key gene conferred the acetoin production. This was the first reported case of C4 chemical production by a thermophilic acetogen. Strain Y72 has been reported to be less stringent in the restriction-modification system, providing high transformation efficiency. A simplified procedure without customized DNA premodification may be helpful in accelerating the metabolic engineering of thermophilic acetogens. There are a few reports concerning acetoin production by thermophiles, but not by thermophilic acetogens that utilize gaseous substrates (Xiao et al., 2012; Sheng et al., 2023).

Two strains for acetoin production were constructed by tuning carbon flux. Based on the design of the metabolic pathways (Figure 1), certain levels of acetate production are needed to maintain metabolism in terms of the metabolic redox state and energy supply. The Y72-*pduL2*::*bsALDC* strain showed higher acetoin production than the Y72-*pyrF*::*bsALDC* strain, demonstrating that knockout of a gene in the acetate pathway was effective in improving acetoin production.

Varying the fermentation temperature and sugar substrate (hexose to pentose) maintained acetoin production itself; however, these changes significantly affected the yield. The differences could be derived from the responsible enzymatic activities, levels of gene expression, or metabolic redox state, in addition to the original metabolic features of *M. thermoacetica* Y72. For example, both key enzymatic activities, PduL1 for acetate and bsALDC for acetoin, were higher at 60°C than at 55°C *in vitro* (Breitkopf et al., 2016; Jia et al., 2017). Furthermore, PduL1 activity was more enhanced to a greater extent by a temperature shift (Breitkopf et al., 2016). These differences may have contributed to different culture profiles at different temperatures. In addition, it has been reported that the NAD(P)/NAD(P)H ratio affects distribution of the metabolic flux during acetoin and byproducts (Bao et al., 2015). The difference due to sugar type could be derived from this redox state (Bao et al., 2015). Some of these factors may affect the consequences of competing chemical productions. In future studies, the involvement of these multiple factors should be investigated to optimize the acetoin production from sugars.

Acetoin production from gaseous substrates has been demonstrated using syngas and CO_2_+H_2_ gas. In both cases, small amounts of acetoin were produced. One reason for this low production may be the utilization of pyruvate as a key intermediate. Whereas the conversion of acetyl-CoA to acetate provides ATP, the conversion of acetyl-CoA to pyruvate requires an energy source (Figure 1B). Because the energy provision from gaseous substrates is low (Schuchmann and Muller, 2014; Debabov, 2021), cellular growth or anabolic metabolism in acetogens is limited; hence, pyruvate production is limited. Energy shortage was evident in the culture profile, especially when H_2_ was solely provided as the energy source, with inactive cell growth and excess formate accumulation. A drastic strategy is necessary for higher acetoin production through enhanced pyruvate production accompanied by sufficient energy in gaseous substrates. However, the detectable level of acetoin indicated that the redox state of metabolism was not as heavily impaired as that of the ethanol-producing Mt-Δ*pduL2*::*aldh* strain (Takemura et al., 2021). The Mt-Δ*pduL2*::*aldh* strain had a *pduL2* knockout and enhanced the expression of aldehyde dehydrogenase for ethanol production. However, it only produced formate and acetate in CO_2_+H_2_ gas.

## 5 Conclusion

Acetoin production by a thermophilic acetogen was demonstrated by successful genetic engineering of *M. thermoacetica* Y72 using a simplified procedure. The engineered strains produced acetoin from diverse carbon sources, and the culture profiles indicated metabolic bottlenecks that warrant further investigation. Notably, the acetoin productivity needs to be far more enhanced for economically viable production. Nevertheless, this study is valued in a concrete progress for the development of a microbial chassis of thermophilic acetogens for the carbon conversion, such as sugars and carbon-rich gases.

## Data Availability

The original contributions presented in the study are included in the article/Supplementary Material, further inquiries can be directed to the corresponding author.

## References

[B1] Abdel-Banat B. M. Hoshida H. Ano A. Nonklang S. Akada R. (2010). High-temperature fermentation: how can processes for ethanol production at high temperatures become superior to the traditional process using mesophilic yeast? Appl. Microbiol. Biotechnol. 85, 861–867. 10.1007/s00253-009-2248-5 19820925

[B2] Bao T. Zhang X. Zhao X. Rao Z. Yang T. Yang S. (2015). Regulation of the NADH pool and NADH/NADPH ratio redistributes acetoin and 2,3-butanediol proportion in *Bacillus subtilis* . Biotechnol. J. 10, 1298–1306. 10.1002/biot.201400577 26129872

[B3] Bauchop T. Elsden S. R. (1960). The growth of micro-organisms in relation to their energy supply. J. Gen. Microbiol. 23, 457–469. 10.1099/00221287-23-3-457 13687855

[B4] Bourgade B. Minton N. P. Islam M. A. (2021). Genetic and metabolic engineering challenges of C1-gas fermenting acetogenic chassis organisms. FEMS Microbiol. Rev. 45, fuab008. 10.1093/femsre/fuab008 33595667 PMC8351756

[B5] Breitkopf R. Uhlig R. Drenckhan T. Fischer R. J. (2016). Two propanediol utilization-like proteins of *Moorella thermoacetica* with phosphotransacetylase activity. Extremophiles 20, 653–661. 10.1007/s00792-016-0854-6 27338272

[B6] Debabov V. G. (2021). Acetogens: biochemistry, bioenergetics, genetics, and biotechnological potential. Microbiology 90, 273–297. 10.1134/s0026261721030024

[B7] Drake H. L. Daniel S. L. (2004). Physiology of the thermophilic acetogen *Moorella thermoacetica* . Res. Microbiol. 155, 869–883. 10.1016/j.resmic.2004.10.002 15630808

[B8] Hu P. Chakraborty S. Kumar A. Woolston B. Liu H. Emerson D. (2016). Integrated bioprocess for conversion of gaseous substrates to liquids. Proc. Natl. Acad. Sci. U. S. A. 113, 3773–3778. 10.1073/pnas.1516867113 26951649 PMC4833252

[B9] Iwasaki Y. Kita A. Sakai S. Takaoka K. Yano S. Tajima T. (2013). Engineering of a functional thermostable kanamycin resistance marker for use in *Moorella thermoacetica* ATCC39073. FEMS Microbiol. Lett. 343, 8–12. 10.1111/1574-6968.12113 23448690

[B10] Iwasaki Y. Kita A. Yoshida K. Tajima T. Yano S. Shou T. (2017). Homolactic acid fermentation by the genetically engineered thermophilic homoacetogen *Moorella thermoacetica* ATCC 39073. Appl. Environ. Microbiol. 83, e00247. 10.1128/aem.00247-17 28159797 PMC5377493

[B11] Jia X. Liu Y. Han Y. (2017). A thermophilic cell-free cascade enzymatic reaction for acetoin synthesis from pyruvate. Sci. Rep. 7, 4333. 10.1038/s41598-017-04684-8 28659601 PMC5489476

[B12] Kato J. Matsuo T. Takemura K. Kato S. Fujii T. Wada K. (2024). Isopropanol production via the thermophilic bioconversion of sugars and syngas using metabolically engineered *Moorella thermoacetica* . Biotechnol. Biofuels Bioprod. 17, 13. 10.1186/s13068-024-02460-1 38281982 PMC10823632

[B13] Kato J. Takemura K. Kato S. Fujii T. Wada K. Iwasaki Y. (2021). Metabolic engineering of Moorella thermoacetica for thermophilic bioconversion of gaseous substrates to a volatile chemical. Amb. Express 11, 59. 10.1186/s13568-021-01220-w 33891189 PMC8065083

[B14] Kita A. Iwasaki Y. Sakai S. Okuto S. Takaoka K. Suzuki T. (2013a). Development of genetic transformation and heterologous expression system in carboxydotrophic thermophilic acetogen *Moorella thermoacetica* . J. Biosci. Bioeng. 115, 347–352. 10.1016/j.jbiosc.2012.10.013 23177215

[B15] Kita A. Iwasaki Y. Yano S. Nakashimada Y. Hoshino T. Murakami K. (2013b). Isolation of thermophilic acetogens and transformation of them with the *pyrF* and *kan^r^ * genes. Biosci. Biotechnol. Biochem. 77, 301–306. 10.1271/bbb.120720 23391907

[B16] Maina S. Prabhu A. A. Vivek N. Vlysidis A. Koutinas A. Kumar V. (2022). Prospects on bio-based 2,3-butanediol and acetoin production: recent progress and advances. Biotechnol. Adv. 54, 107783. 10.1016/j.biotechadv.2021.107783 34098005

[B17] Rahayu F. Kawai Y. Iwasaki Y. Yoshida K. Kita A. Tajima T. (2017). Thermophilic ethanol fermentation from lignocellulose hydrolysate by genetically engineered *Moorella thermoacetica* . Bioresour. Technol. 245, 1393–1399. 10.1016/j.biortech.2017.05.146 28583404

[B18] Redl S. Poehlein A. Esser C. Bengelsdorf F. R. Jensen T. O. Jendresen C. B. (2019). Genome-based comparison of all species of the genus *Moorella*, and status of the species *Moorella thermoacetica* and *Moorella thermoautotrophica* . Front. Microbiol. 10, 3070. 10.3389/fmicb.2019.03070 32010113 PMC6978639

[B19] Redl S. Sukumara S. Ploeger T. Wu L. Olshoj Jensen T. Nielsen A. T. (2017). Thermodynamics and economic feasibility of acetone production from syngas using the thermophilic production host *Moorella thermoacetica* . Biotechnol. Biofuels 10, 150. 10.1186/s13068-017-0827-8 28616074 PMC5469130

[B20] Schuchmann K. Muller V. (2014). Autotrophy at the thermodynamic limit of life: a model for energy conservation in acetogenic bacteria. Nat. Rev. Microbiol. 12, 809–821. 10.1038/nrmicro3365 25383604

[B21] Sheng L. Madika A. Lau M. S. H. Zhang Y. Minton N. P. (2023). Metabolic engineering for the production of acetoin and 2,3-butanediol at elevated temperature in *Parageobacillus thermoglucosidasius* NCIMB 11955. Front. Bioeng. Biotechnol. 11, 1191079. 10.3389/fbioe.2023.1191079 37200846 PMC10185769

[B22] Takemura K. Kato J. Kato S. Fujii T. Wada K. Iwasaki Y. (2021). Autotrophic growth and ethanol production enabled by diverting acetate flux in the metabolically engineered *Moorella thermoacetica* . J. Biosci. Bioeng. 132, 569–574. 10.1016/j.jbiosc.2021.08.005 34518108

[B23] Tanner R. S. (1989). Monitoring sulfate-reducing bacteria - comparison of enumeration media. J. Microbiol. Methods 10, 83–90. 10.1016/0167-7012(89)90004-3

[B24] Tanner R. S. Miller L. M. Yang D. (1993). *Clostridium ljungdahlii* sp. nov., an acetogenic species in clostridial rRNA homology group I. Int. J. Syst. Bacteriol. 43, 232–236. 10.1099/00207713-43-2-232 7684239

[B25] Taylor M. P. Eley K. L. Martin S. Tuffin M. I. Burton S. G. Cowan D. A. (2009). Thermophilic ethanologenesis: future prospects for second-generation bioethanol production. Trends Biotechnol. 27, 398–405. 10.1016/j.tibtech.2009.03.006 19481826

[B26] Tsukahara K. Kita A. Nakashimada Y. Hoshino T. Murakami K. (2014). Genome-guided analysis of transformation efficiency and carbon dioxide assimilation by *Moorella thermoacetica* Y72. Gene 535, 150–155. 10.1016/j.gene.2013.11.045 24316126

[B27] Werpy T. A. Petersen G. E. Aden A. Bozell J. J. Holladay J. E. White J. F. (2004). Top value added chemicals from biomass: volume I - results of screening for potential candidates from sugars and synthesis gas. Tech. Rep. Top Value Added Chem. Biomass 1. 10.2172/15008859

[B28] Xiao Z. Wang X. Huang Y. Huo F. Zhu X. Xi L. (2012). Thermophilic fermentation of acetoin and 2,3-butanediol by a novel *Geobacillus* strain. Biotechnol. Biofuels 5, 88. 10.1186/1754-6834-5-88 23217110 PMC3538569

