# Supplementary material for: Genetic engineering of a thermophilic acetogen, Moorella thermoacetica Y72, to enable acetoin production
Source: Front Bioeng Biotechnol. 2024 May 15;12:1398467. doi: 10.3389/fbioe.2024.1398467 (PMC11133584; doi:10.3389/fbioe.2024.1398467)
Supplement: Supplementary file 1 [file Table1.DOCX]

Table S1

Primers used in this study

Primer name Sequence

JK109 AAGGAGGAGTACATAATGAAGCGGGAGTCCAAC

JK110 ATGAAAGCAGGCCGATTATTCGGGGCTGCCTTC

JK52 TCGGCCTGCTTTCATGCTTG

JK71 TATGTACTCCTCCTTATATTTATTGTAACGGC

bsALDC_FW  AAGGAGGAGTACATAATGAAGCGGGAGTCCAAC

bsALDC_RV  ATGAAAGCAGGCCGATCAAGCATGAAAGCAGGCC

Vec_FW     TCGGCCTGCTTTCATGCTTGATAAT

Vec_RV     TATGTACTCCTCCTTATATTTATTG

pduL2_check_FW CCTCTGATAAGTAATTGCAACGGCC

pduL2_check_Rv CATTAACCAGAGCACCTCCTTATAC

pyrF72_upF GTCCTCAACACCCTGACC

pyrF_downR TCTTCCCAGGTCCTGTAGG
